# The role of neurologists in the era of cancer immunotherapy: Focus on CAR T-cell therapy and immune checkpoint inhibitors

**DOI:** 10.3389/fneur.2022.936141

**Published:** 2022-07-19

**Authors:** Umberto Pensato, Maria Guarino, Lorenzo Muccioli

**Affiliations:** ^1^Department of Neurology, IRCCS Humanitas Research Hospital, Milan, Italy; ^2^IRCCS Istituto delle Scienze Neurologiche di Bologna, Bologna, Italy; ^3^Department of Biomedical and Neuromotor Sciences, University of Bologna, Bologna, Italy

**Keywords:** haematological cancers, cytokine release syndrome, cytokine storm-associated encephalopathy (CySE), paraneoplastic neurological syndromes, neurotoxicity, lymphoma, neuroimmunology, cytokine-mediated neuroinflammation

## Abstract

Cancer immunotherapy represents a novel anticancer strategy that acts directly on the immune system, promoting its activation toward cancer cells to enhance its natural ability to fight cancer. Among various treatments currently used or investigated, chimeric antigen receptors (CAR) T-cell therapy and immune checkpoint inhibitors (ICIs) have consistently proven their efficacy. These innovations are progressively improving the standard of care in cancer treatment, yet they are hampered by novel neurological adverse events, attributing to neurologists a key role in the multidisciplinary oncological team. Indeed, neurotoxicity may develop in up to 77% of patients who received CAR T-cell therapy and usually presents with encephalopathy characterized by a predominant frontal lobe dysfunction. This neurotoxicity is related to cytokine release syndrome, a systemic hyperinflammatory condition triggered by CAR T-cells. On the other hand, following treatment with ICIs, unrestrained T-cells may lead to central and peripheral neurological disorders by antigen-directed autoimmunity. Notably, biological and clinical similarities have been underlined between neurotoxicity related to CAR T-cell therapy and neurological manifestations of cytokine storms (e.g. COVID-19-related encephalopathy), as well as between a subgroup of ICI-related neurological adverse events and paraneoplastic neurological syndromes. Therefore, these cancer immunotherapy-related neurological syndromes may provide an unprecedented, perhaps transitory, opportunity to shed light on the underlying pathogenic mechanisms of a wide spectrum of neurological syndromes and to push forward our knowledge in neuroimmunology.

## Introduction

Nowadays, we are living the long-awaited revolution in cancer treatment, as immunotherapies have expanded our armamentarium to fight cancer, especially in the last decade (1). These welcome innovations are progressively improving the standard of care in both solid and hematological cancer treatment, yet they are hampered by novel adverse events, including neurotoxicity, gaining complexity in the management of oncological patients (2, 3). Hence, neurologists have to face new challenges and have been acquiring a critical role in the multidisciplinary cancer team as, in a subset of patients, treatment-related neurotoxicity may represent the limiting factor of cancer immunotherapies. Additionally, these novel adverse events have further expanded the field of neuro-oncology, which was previously limited to the management of nervous system neoplasms or metastasis and disorders directly related to malignancies, namely paraneoplastic neurological syndromes (PNS).

Thousands of clinical trials investigating a multitude of different therapies have been established worldwide, including cellular therapies and checkpoint inhibitors [ongoing clinical trials identified on clinicaltrial.gov on Apr-25-2022: chimeric antigen receptor T-cell (CAR-T) therapy, *n* = 1,131, immune checkpoint inhibitors (ICI), *n* = 2,548], and neurologists' collaboration is usually required in each participating site. This represents an exciting challenge and opportunity for neurologists who will cope with poorly described neurological syndromes, utterly different from those related to traditional chemotherapy, which still require to be dissected and characterized in-depth (4). Additionally, neurological complications of cancer immunotherapy may represent a framework model to shed light on other recognized, yet not fully understood immune-mediated neurological syndromes. Furthermore, neurologists will have to cope with a management conundrum, since a balance between anticancer and anti-neurotoxicity actions may be challenging to achieve. Indeed, treatments specific for neurotoxicity related to an overactivated immune system, such as corticosteroids, may interfere with the anticancer actions of immunotherapy; on the other hand, stopping prematurely neurotoxic immunotherapy, such as immune checkpoint inhibitors (ICIs), evidently reduces the chance of defeating cancer.

## Activation immunotherapy

Activation immunotherapy represents a novel anticancer strategy that acts directly on the immune system, promoting its activation toward cancer cells to enhance its natural ability to fight cancer (1). This arguably represents one of the most relevant achievements in medicine in the last decades, as confirmed by the 2018 Nobel prize awarded to the discoveries in basic science allowing the development of checkpoint inhibitor therapies (5).

A wide spectrum of different techniques to enhance the immune system is being currently investigated. T-cells adoptive therapies and ICIs have consistently proven their efficacy and are routinely administered to patients with refractory malignancies, having become one of the most common causes of cancer treatment-related neurotoxicity encountered in clinical practice (4).

We will briefly discuss the mechanism of action, therapeutic use, and neurotoxicity of these two treatments as illustrative models to outline the neurologists' potential “honors and burdens” in the era of cancer immunotherapy.

## CAR T-cell therapy-related neurotoxicity and cytokine storm-associated encephalopathy

CAR T-cell therapy is a novel treatment for refractory hematological cancers, which has demonstrated complete remission responses in 33%−57% of patients (6). Autologous T-cells are isolated and genetically engineered to redirect their cytotoxicity specifically toward tumor cells. Subsequently, the cells are expanded and reinfused after the patient has received preconditioning chemotherapy (6). While several different tumor antigens have been investigated as targets of CAR T-cells, anti-CD19 CAR T-cells are the most advanced in clinical development and are currently approved by the U.S. Food and Drug Administration (FDA) for various B-cells malignancies (Table 1). Additionally, attempts to expand CAR T-cells clinical use to treat solid cancers, autoimmune disorders, and infections are ongoing (7, 8). Toxicities are mainly driven by the activation of infused CAR T-cells, which lead to exaggerated cytokine-mediated inflammatory responses, namely cytokine release syndrome (CRS), that usually occur within 48 h since infusion and may lead to multi-organ failure (2). Immune effector cell-associated neurotoxicity syndrome (ICANS) is another recurrent complication with a remarkable association with CRS, yet it usually develops a few days later and is therefore considered as a distinct condition (2). Strong evidence supports a cytokine-mediated neuroinflammatory process as the underlying mechanism, where the peripheral inflammation leads to blood-brain barrier disruption and endothelial dysfunction, resulting in astrocyte and microglia activation (9). Accordingly, neurotoxicity grade correlates with CSF cytokine levels and not with CSF CAR T-cells levels (9). The incidence of neurotoxicity following CAR T-cells immunotherapy is highly heterogeneous, mainly depending on CAR T-cells products, yet it may occur in up to 77% of infused patients (10). Clinical and instrumental findings of neurotoxicity have been poorly characterized thus far, yet some recurrent features have emerged. Encephalopathy, variably accompanied by early language disturbances, such as expressive aphasia and dysgraphia, and frontal lobe dysfunction represent recurrent clinical features (2, 9, 11, 12). The disease has a heterogeneous course, and while some patients experience mild neurological manifestations with spontaneous recovery, a subgroup progresses to develop seizures, akinetic mutism, and coma, potentially leading to death (2, 13, 14). Notably, clinical and investigative similarities have been underlined between neurological manifestations of different cytokine storm disorders, mostly COVID-19, and CAR T-cell therapy-related neurotoxicity (11, 15–17). Indeed, COVID-19-related encephalopathy/encephalitis is characterized by a frontal-predominant electro-clinical dysfunction associated with elevated CSF cytokines levels, together with several other shared features (11, 16, 18). Accordingly, these neurological disorders may represent an etiological coherent disease continuum, sharing cytokine storm as a common denominator.

**Table 1:** Molecular mechanisms, targeted cancers and neurological syndromes of different immunotherapies^*^.

**Immunotherapies**	**Molecular target: names (year of FDA approval)**	**Targeted cancers (FDA approved)**	**Serious adverse neurological events**
CAR T-cell therapy	• Anti-CD19: Tisagenlecleucel (2017), axicabtagene cioleucel (2017), brexucabtagene autoleucel (2020), lisocabtagene maraleucel (2021) • Anti-BCMA: idecabtagene vicleucel (2021)	• Acute lymphoblastic leukemia • Diffuse large B-cell lymphoma • Primary mediastinal B-cell lymphoma • High-grade B-cell lymphoma • Follicular lymphoma • Mantle cell lymphoma • Multiple myeloma	Immune effector cell-associated neurotoxicity syndrome (ICANS) • Frontal predominant encephalopathy • Language disturbances • Dysgraphia • Seizures • Impaired consciousness • Akinetic mutism
Immune checkpoint inhibitors	• Anti-CTLA-4: Ipilimumab (2011) • Anti-PD-1: Nivolumab (2014), Pembrolizumab (2014), Cemiplimab (2018) • Anti-PD-L1: Atezolizumab (2016), Avelumab (2017), Durvalumab (2017)	• Skin (Metastatic melanoma, squamous cell and basal cell carcinoma) • Uro-genital (renal cell carcinoma, urothelial carcinoma, bladder cancer, cervical cancer, endometrial cancer • Lungs ( small and non-small cell lung cancers, colorectal cancer, malignant pleural mesothelioma) • Gastro-intestinal (hepatoceullar, esophageal, gastric and colorectal carcinoma) • Breast cancer • Hodgkin's lymphoma • Head and neck cancer • Merkel cell carcinoma	• Meningitis • Encephalitis (e.g. limbic encephalitis, cerebellitis) • Demyelinating syndromes (e.g. optic neuritis, transverse myelitis) • CNS vasculitis • Neuropathy (e.g. CIDP, Guillan-Barrè syndromes) • Neuromuscular junction disorders (e.g. myasthenia gravis, LEMS) • Myopathy (e.g. immune-mediated necrotizing myopathy)

* *The present table is not meant to be an exhaustive list of cancer subtypes eligible for immunotherapies or neurological adverse events related to immunotherapies, yet it's an attempt to give the readers a general framework*.

*FDA, U.S. Food and drug administration; CAR, chimeric antigen receptor; BCMA, B-cell maturation antigen; CTLA-4, cytotoxic T-lymphocyte antigen 4; PD-1, programmed death protein 1; PD-L1, programmed death-ligand 1; CIDP, chronic inflammatory demyelinating polyneuropathy; LEMS, Lambert-Eaton myasthenic syndrome*.

## Immune checkpoint inhibitors therapy-related neurotoxicity and paraneoplastic neurological syndromes

ICIs are monoclonal antibodies that prevent the tumor microenvironment from inducing immunosuppression by blocking regulatory checkpoints, eventually restoring T-cells responses against cancer. Currently, there are seven FDA-approved ICIs for several cancer subtypes (Table 1). Unfortunately, up to 12% of patients treated with ICIs develop central or, more frequently, peripheral neurological disorders (3). The underlying mechanisms seem utterly different from those involved in ICANS, where encephalopathy is associated with the cytokine storm (11, 19). Indeed, following treatment with ICIs, unrestrained T-cells may result in antigen-directed autoimmunity, either antibody- or T cell-mediated. Interestingly, several lines of evidence, including clinical phenotypes and serum/CSF autoantibody signature, substantiate the concept that these neurological complications represent either unleashed autoimmune or, less frequently, paraneoplastic neurological syndromes (PNS) (20, 21). PNS comprise a heterogeneous group of immune-mediated neurological disorders, which may represent the heralding clinical presentation of a tumor (22). These disorders likely result from the ectopic expression of neural proteins in the tumor cells (onconeural antigens), leading to a misdirected immune response against the nervous system (21). Accordingly, patients display a distinctive signature of autoantibodies directed against cell surface or intracellular neural proteins. The former are considered directly pathogenic, reflecting antibody-mediated autoimmunity, whereas the latter is likely an epiphenomenon of predominant T-cell-mediated autoimmunity (22). PNS are rare, occurring in <1% of cancer patients, yet, beyond cancer histological subtypes, their risk factors remain largely unknown (21). Interestingly, preliminary evidence suggests that mutated onconeural antigens expressed by tumor cells and specific HLA haplotypes are potential promoting mechanisms (21). Therefore, ICIs exposure arguably represents a major risk factor for developing PNS and provide the unique possibility to perform serial evaluations of the immune profile of cancer patients before and after developing neurological manifestations, potentially allowing to shed light on PNS underlying pathogenic mechanisms.

## Discussion

Assessment and management of neurotoxicity related to cancer immunotherapy is a rapidly evolving field at the intersection of neuroimmunology and neuro-oncology, yet it requires skills in most neurological subspecialties, as well as in oncology and clinical immunology. As awareness of these neurological disorders increases, training neurologists to recognize and manage them is becoming a major need. Currently, education opportunities are mostly limited to large oncology clinics where these novel cancer immunotherapies are routinely administered, yet a more widespread use is expected in the very next future. To this end, dedicated fellowship training, perhaps within neuro-oncology and neuroimmunology programs, will be necessary to fill this gap.

Cancer immunotherapies will continue to evolve, and so will their neurological adverse events. Nonetheless, the cross-sectional knowledge acquired from these adverse events will arguably come in handy. Indeed, biological and clinical similarities have been underlined between neurotoxicity related to CAR T-cell therapy and neurological manifestations of cytokine storms (e.g. COVID-19-related encephalopathy), as well as between a subgroup of ICI-related neurological adverse events and paraneoplastic neurological syndromes. Therefore, these cancer immunotherapy-related neurological syndromes may provide an unprecedented, perhaps transitory, opportunity to shed light on the underlying pathogenic mechanisms of a wide spectrum of neurological syndromes and to push forward our knowledge in neuroimmunology.

## Data availability statement

The original contributions presented in the study are included in the article/supplementary material, further inquiries can be directed to the corresponding author.

## Author contributions

UP: drafting of the manuscript for content, including medical writing for content, study concept or design, and analysis or interpretation of data. MG and LM: revision of the manuscript for content, study concept or design, and analysis or interpretation of data. All authors contributed to the article and approved the submitted version.

## Conflict of interest

The authors declare that the research was conducted in the absence of any commercial or financial relationships that could be construed as a potential conflict of interest.

## Publisher's note

All claims expressed in this article are solely those of the authors and do not necessarily represent those of their affiliated organizations, or those of the publisher, the editors and the reviewers. Any product that may be evaluated in this article, or claim that may be made by its manufacturer, is not guaranteed or endorsed by the publisher.
